# COVID-19 Vaccine Hesitancy and Associated Factors among Diabetes Patients: A Cross-Sectional Survey in Changzhi, Shanxi, China

**DOI:** 10.3390/vaccines10010129

**Published:** 2022-01-17

**Authors:** Ying Wang, Lingrui Duan, Mufan Li, Jiayu Wang, Jianzhou Yang, Congying Song, Jing Li, Jinsheng Wang, Jiantao Jia, Junjie Xu

**Affiliations:** 1 School of Epidemiology and Public Health, Shanxi Medical University, Taiyuan 032000, China; wangying@b.sxmu.edu.cn (Y.W.); dlr0875@b.sxmu.edu.cn (L.D.); lmf98@b.sxmu.edu.cn (M.L.); JiaYu12@b.sxmu.edu.cn (J.W.); 2 Department of Preventive Medicine, Changzhi Medical College, Changzhi 046000, China; 3 Clinical Research Academy, Peking University Shenzhen Hospital, Peking University, Shenzhen 518000, China; songcongying@pkuszh.com; 4 Renal Division, Peking University Shenzhen Hospital, Peking University, Shenzhen 518000, China; lijingp0677@pkuszh.com; 5 Department of Pathology and Basic Medicine, Changzhi Medical College, Changzhi 046000, China; jshwang@czmc.edu.cn; 6 Department of Pathophysiology and Basic Medicine, Changzhi Medical College, Changzhi 046000, China; jiajt985@czmc.edu.cn

**Keywords:** diabetes, SARS-CoV-2 vaccine, health belief model, vaccine hesitancy

## Abstract

Patients with diabetes are more susceptible to severe acute respiratory syndrome-associated coronavirus (SARS-CoV)-2 infection, but vaccine hesitancy is a problem in this population. We investigated the prevalence of SARS-CoV-2 vaccine hesitancy among diabetes patients in China through a cross-sectional survey from April and August 2021 using a questionnaire administered to patients at two hospitals affiliated with Changzhi Medical College (Shanxi, China). The health belief model (HBM) is used examining factors influencing vaccine hesitancy. After adjusting for potential confounders, a multivariate logistic regression model was used to analyze correlations between vaccine hesitancy and associated factors. Of the 483 participants, 56.4% (273/483) had vaccine hesitancy, including 58.2% (159/273) who were unsure of being vaccinated and 41.8% (114/273) who were unwilling. Although patients considered SARS-CoV-2 infection to be serious (adjusted odds ratio [aOR] = 3.90, 95% confidence interval [CI]: 2.36–6.42; *p* < 0.001), they had concerns about vaccine safety (aOR = 3.05, 95% CI: 1.89–4.91; *p* < 0.001). Relatives’ vaccination status did not influence participants’ willingness to be vaccinated (aOR = 2.43, 95% CI: 1.39–4.25; *p* < 0.001). Disagreement with physicians’ view that vaccination can reduce SARS-CoV-2 infection risk was independently correlated with vaccine hesitancy (aOR = 2.25, 95% CI: 1.28–3.95; *p* < 0.001). Diabetes patients in China need to be educated on SARS-CoV-2 vaccine safety and protective effects to increase the vaccination rate in this population.

## 1. Introduction

Patients with diabetes and associated complications are more susceptible to infection with severe acute respiratory syndrome-associated coronavirus (SARS-CoV-2), the causative agent of coronavirus disease 2019 (COVID-19), and have a high risk of severe COVID-19-associated morbidity and mortality [1,2,3]. Data from the Centers for Disease Control and Prevention indicate that diabetic patients have 5- and 3-fold higher risks of contracting SARS-CoV-2 and dying from COVID-19, respectively, compared to individuals without diabetes [4,5,6]. This may be due to acute or chronic complications of diabetes that impact immunity [7,8,9]. Diabetes is among the fastest growing global health emergencies of the 21st century, with an estimated 643 million people (11.3% of the population) living with diabetes by 2030. China holds the highest prevalence of diabetes in the world, and it is estimated that there will be 14.086 million diabetic patients in 2021 [10]. While diabetic patients are more susceptible to infection with severe acute respiratory SARS-CoV-2, there are few reports about COVID-19 vaccination safety and immunological reactions, and there is no report about the willingness of COVID-19 vaccination in the diabetic group. Despite doctors’ recommendations, more proportion of the people with diabetes is reluctant to get vaccinated, compared with the general population (14.2–29.0% vs. 8.7%) [11,12,13,14]. As the number of people suffering from diabetes continues to increase, it will bring a huge burden to China’s health and economy. Despite studies indicating increased hesitation to SARS-CoV-2 immunization in chronic illness groups, diabetic individuals are an essential component and typical of the chronic disease community [15]. However, there are various kinds of chronic patients, and it is extremely difficult to recruit all these kinds of patients in one study. Therefore, we conducted this study among diabetic patients to investigate their willingness to vaccinate.

The SARS-CoV-2 vaccine plays a critical role in overcoming the COVID-19 pandemic. A calculation model simulation study of SARS CoV-2 vaccine efficacy showed that the vaccine has to have an efficacy of at least 70% to prevent an epidemic and of at least 80% to largely extinguish an epidemic without any other measures [16]. Most phase 3 clinical trials of SARS-CoV-2 vaccines have not included patients with comorbidities including diabetes; as such, there is a lack of real-world evidence on vaccine side effects and protective effects in this population and the existing data are inconsistent. A study on the antibody levels of diabetic and non-diabetic individuals 3 weeks after immunization with two doses of BNT162b2 mRNA COVID-19 vaccination showed that the neutralizing antibody produced by diabetic patients is 4.42% lower than that of non-diabetic patients [17]. Similarly, a study from Turkey showed that diabetes patients produced lower levels of antibodies four weeks after immunization with two doses of the SARS-CoV-2 vaccine [18].

Current guidelines or recommendations for SARS-CoV-2 vaccination in diabetes patients also vary across countries. The World Health Organization, Europe, United States, and South Korea recommend vaccination [19,20,21,22,23,24], but Chinese guidelines exclude individuals with chronic diseases that are poor-controlled by medication [25]. The lack of direct evidence on the benefits of vaccination has led vaccine hesitancy among diabetes patients, with rates of 14.2% in Italy [11], 29.0% in Saudi Arabia [12], and 24.7% in Malaysia [26]. Previous studies on vaccine hesitancy in patients with diabetes have focused on high-income countries, and there is limited information on prevailing attitudes in low- and middle-income countries. A recent survey conducted in Uganda found that 29.90% of patients with chronic illnesses, including diabetes, were hesitant to receive the SARS-CoV-2 vaccine [27]. The main reasons for vaccine hesitancy among diabetes patients are concerns regarding vaccination side effects and uncertainty about vaccine composition [11,12,26]. To date, no studies have used a theory-based analysis to investigate the reasons for vaccine hesitancy.

The Health Belief Model (HBM) is a health education model that includes information in six dimensions, including Perceived susceptibility, Perceived severity, Perceived benefits, Perceived barriers, self-efficacy to engage in a behavior, and Action clues [28]. The HBM has been used to measure population’s attitudes towards to vaccination against papillomavirus (HPV), influenza, H1N1, and SARS CoV-2 virus [29,30,31,32,33]. The measured outcomes are also helpful to analyze behavior-related influencing factors and to change the vaccination behaviors, cognitions, attitudes, and beliefs of individuals through health education.

In China, the main vaccines used for SARS-CoV-2 are inactivated vaccines. As there are no data on the willingness of diabetes patients to receive the vaccine in China, we carried out a cross-sectional survey based on the HBM to determine the prevalence of SARS-CoV-2 vaccine hesitancy in this population, as well as the associated factors.

## 2. Materials and Methods

### 2.1. Study Design

In this cross-sectional study conducted from April to August 2021, a questionnaire was administered through face-to-face interviews to diabetes patients hospitalized at the endocrinology department of Heping and Heji Hospitals, Changzhi Medical College (Changzhi, Shanxi, China). Information on sociodemographic characteristics, lifestyle, and disease-related conditions and pertaining to SARS-CoV-2 vaccination, such as attitudes toward vaccination and potential reasons for vaccine hesitancy, was collected.

The questionnaire was prepared by two professors of epidemiology and a clinically experienced endocrinologist after a review of the literature on the willingness to receive the SARS-CoV-2 vaccine in China and other countries. The questionnaire included three major items: (1) demographic information and health status; (2) perceived risk of SARS-CoV-2 infection and knowledge of SARS-CoV-2 vaccines; and (3) willingness to receive a SARS-CoV-2 vaccine.

Demographic information, such as sex, age, ethnicity, education level, occupation, health insurance, diabetes prevalence, and family history, along with the current health status of diabetes patients, including other chronic diseases, diabetes complications, and the extent of glycemic control, were recorded.

The primary outcome of the study was participants’ attitude toward future SARS-CoV-2 vaccination, with “Willing to be vaccinated”, “Unwilling to be vaccinated”, and “Unsure” as possible responses. Based on a previous survey [34], responses of “Unwilling to be vaccinated” and “Unsure” were classified as hesitancy to receive the SARS-CoV-2 vaccine.

We used the HBM to assess patients’ hesitancy to receive the SARS-CoV-2 vaccine. The model included the following information: (1) Perceived susceptibility (“The risk of acquiring SARS CoV-2 is high”), (2) Perceived severity (“The SARS CoV-2 syndrome is severe”), (3) Perceived benefits (“Vaccination reduces the risk of infection”, ”Vaccination reduces the risk of transmission to other people”, and “Vaccination is good for yourself and others”), (4) Perceived barriers (“As a person with diabetes, I worried about the safety of the vaccination” and “Worried about side effects of vaccination”), and (5) Action cues (“Relatives’ vaccination action will affect your vaccination behavior”, “Believe in the doctor’s statement that vaccination can reduce the risk of infection”, and “Advice on vaccination from the internet/media”). Dimensions for the above questions were as follows: (1) strongly disagree, (2) disagree, (3) neutral, (4) agree, and (5) strongly agree.

### 2.2. Inclusion and Exclusion Criteria

Eligible participants were patients diagnosed with type 1 or 2 diabetes who were hospitalized in the two participating hospitals during the study period, Age older than 18 years, and voluntarily participated in the survey and provided written, informed consent. Patients who had been diagnosed with a mental illness or had taken medication for a mental illness in the preceding 3 months, had obvious dementia symptoms, or were unable to communicate verbally with the investigators were excluded.

### 2.3. Sample Size Calculation

According to the sample size formula of estimating a total rate parameter through a cross-sectional study, α is the significance level and it is taken as 0.05, Z1-α/2 is taken as 1.96; prior to the formal survey, we derived an estimated hesitation rate for COVID-19 vaccination of 48.3% (29/60) in local diabetic patients through a pilot-survey; furthermore, δ is the allowable error and δ is taken as 0.05. Therefore, the initial minimum sample size required for the study is 384 participants. In addition, based on the consideration of the participant non-response rate (10%) and the qualified rate of questionnaire (90%), the estimated minimum sample size was 474.

### 2.4. Statistical Analysis

All analyses were performed using SPSS v25.0 software (IBM, Armonk, NY, USA). Qualitative data are presented as frequencies and percentages; quantitative variables are presented as mean and standard deviation, and an independent samples t-test was used for analysis. The crude odds ratio (cOR) was obtained using univariate logistic regression model, with SARS-CoV-2 vaccine hesitancy as the dependent variable and baseline characteristics as independent variables. A multivariate analysis was performed for vaccine hesitancy, while the association between the dependent variable and independent variables of interest (i.e., those in the HBM) was assessed with adjusted dominance ratio (aOR) and 95% confidence interval (CI). The aOR was obtained by fitting a one-way logistic regression model that included the independent variable of interest and all baseline characteristics significant at *p* < 0.10.

## 3. Results

### 3.1. Baseline Characteristics

**Figure 1:**
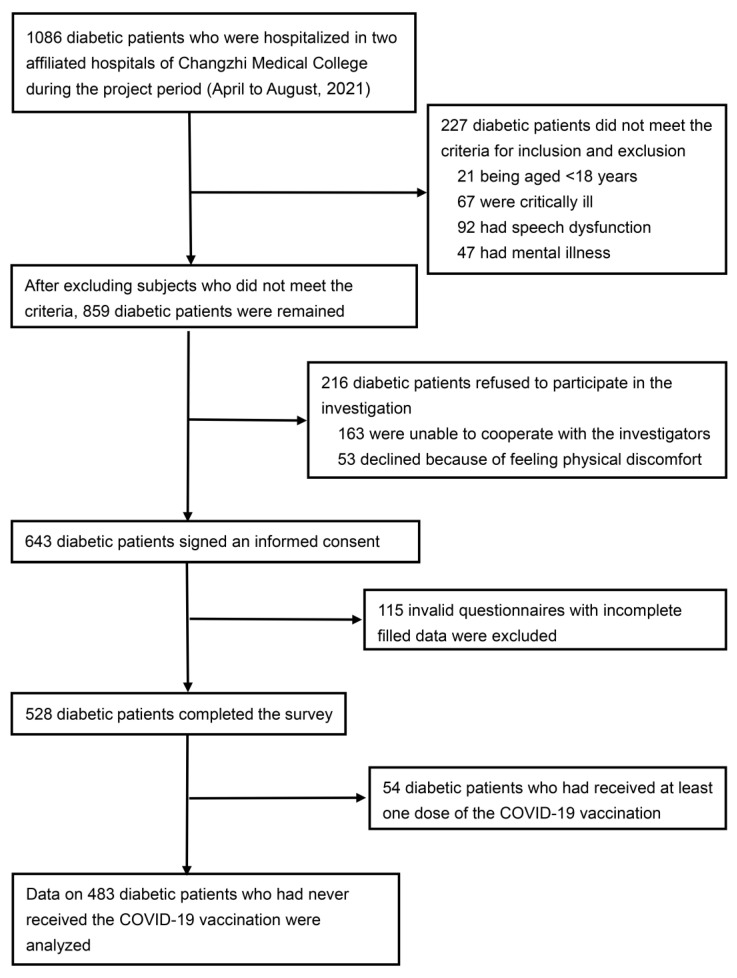
Flowchart of data collection.

We screened 859 diabetes patients; 528 completed the survey and 45 (8.5%) reported having received at least one dose of SARS-CoV-2 vaccine. Statistical analyses were conducted on 483 participants who had not received the SARS-CoV-2 vaccine. The flowchart of patient enrollment is shown in Figure 1.

Of the 483 participants, the male to female and urban to rural resident ratios were nearly 1:1. Most patients were 50–69 years old (*n* = 275, 56.9%), Han Chinese (*n* = 475, 98.3%), had less than high school education (*n* = 288, 59.6%), were married or cohabiting with a partner (*n* = 424, 87.8%), were farmers (*n* = 210, 43.5%), had a per capita monthly income < 2000 yuan (*n* = 205, 42.4%), and urban employee insurance (*n* = 228, 47.2%). In terms of characteristics related to lifestyles and diabetes health status, 70.0% (*n* = 338) of the participants smoked and 74.9% (*n* = 362) drank alcohol, 86.1% (*n* = 416) had other chronic conditions, 67.1% (*n* = 324) had diabetes-associated complications, 64.2% (*n* = 310) had a family history of diabetes, and 30.2% (*n* = 146) had a diabetes diagnosis for over 10 years. Less than 8% had a fasting glucose level >13.9 mmol/L at the most recent testing and nearly 50% reported a postprandial glucose level >11.1 mmol/L (Table 1 and Table 2).

### 3.2. SARS-CoV-2 Vaccination Variables for the HBM

Regarding awareness of SARS-CoV-2, 76.8% of the participants (371/483) did not believe that they were at high risk of SARS-CoV-2 infection, while 31.9% (154/483) considered COVID-19 to be a serious disease; moreover, 66.0% (319/483) thought that vaccination would not reduce their risk of SARS-CoV-2 infection, and 38.5% (186/483) thought that it would not reduce the risk of virus transmission to others. On the other hand, most patients (95.9%, 463/483) thought that vaccination would be beneficial to themselves and others. Regarding perceived barriers, 69.8% of patients (337/483) were concerned about the safety of the SARS-CoV-2 vaccine, with 58.6% (283/483) concerned about side effects. Additionally, 82.4% (398/483) reported that the vaccination status of relatives would not influence their vaccination behavior; moreover, 47.6% (230/483) disagreed with physicians’ view that vaccination reduces the risk of SARS-CoV-2 infection, and 51.8% (250/483) reported no change in their attitude toward vaccination after reading information on the internet or on social media (Table 3).

### 3.3. SARS-CoV-2 Vaccine Hesitancy and Associated Factors

Less than half of the participants (43.6%, 210/483) were willing to receive the SARS-CoV-2 vaccine; among the 56.4% (273/483) who were reluctant to be vaccinated, 58.2% (159/273) were unsure of whether to be vaccinated and 41.8% (114/273) were unwilling to be vaccinated in the future.

In the univariate logistic regression analysis, willingness to receive the SARS-CoV-2 vaccine was lower among diabetes patients with a high school education level. Lifestyle factors such as smoking and health status were unrelated to vaccine hesitancy (Table 1 and Table 2).

After adjusting for differences in baseline characteristics related to social context-, lifestyle-, and disease-related conditions, the perception of COVID-19 as a severe disease (aOR = 3.90, 95% CI: 2.36–6.42; *p* < 0.001), concerns regarding the safety of the SARS-CoV-2 vaccine (aOR = 3.05, 95% CI: 1.89–4.91; *p* < 0. 001), disagreement with the statement that relatives’ vaccination status would influence participants’ vaccination decision (aOR = 2.43, 95% CI: 1.39–4.25; *p* < 0.001), and disagreement with physicians’ view that vaccination reduces the risk of SARS-CoV-2 infection (aOR = 2.25, 95% CI: 1.28–3.95; *p* < 0.001) were associated with vaccine hesitancy (Table 4).

### 3.4. Reasons for Diabetes Patients’ Hesitancy to Receive the SARS-CoV-2 Vaccine

**Figure 2:**
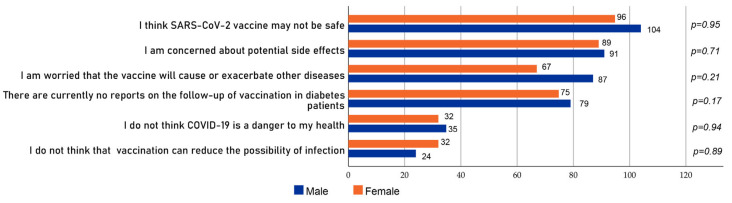
Reasons for SARS-CoV-2 vaccine hesitancy among study participants (*n* = 273).

The main reasons for the reluctance to be vaccinated against SARS-CoV-2 among diabetes patients were as follows: belief that the SARS-CoV-2 vaccine is unsafe (11.84% in males and 12.82% in females, *p* = 0.95); fear of side effects from the vaccine (10.97% in males and 11.22% in females, *p* = 0.71); fear of other adverse reactions after vaccination (8.26% in males and 10.73% in females, *p* = 0.21); no current reports of follow-up after vaccination of diabetes patients (9.25% in males and 9.74% in females, *p* = 0.17); belief that COVID-19 is not dangerous to diabetes patients’ health (3.95% in males and 4.32% in females, *p* = 0.94); and belief that vaccination does not reduce the risk of infection (3.95% in males and 2.96% in females, *p* = 0.89). There were no statistically significant differences between males and females in any of the above reasons (Figure 2).

## 4. Discussion

Vaccination against SARS-CoV-2 can significantly reduce the risk of COVID-19 in patients with chronic diseases including diabetes. In the published literature reports, no significant adverse effects were found in diabetics after receiving the SARS CoV-2 vaccine [35]. Therefore, initiatives to promote SARS-CoV-2 vaccination in this population are needed. The present study is the first to report the prevalence of SARS-CoV-2 vaccine hesitancy among Chinese diabetes patients. We also determined through a questionnaire based on the HBM that action cues were the main factors influencing vaccine hesitancy in our population. These findings provide a basis for targeted education and behavioral interventions to improve SARS-CoV-2 vaccine coverage in at-risk groups and reduce their risk of morbidity and mortality from COVID-19.

The proportion of patients with diabetes who were hesitant to receive the SARS-CoV-2 vaccine (56.4%, 95%CI: 52.0–60.8) was significantly higher than in diabetes patients in higher-income countries, such as Italy (14.2–18.3%) [11,13] and Saudi Arabia (29%) [12], and in patients with various chronic diseases including diabetes in Uganda (29.9%) [27] and Saudi Arabia (48%) [36]. Thus, SARS-CoV-2 vaccine hesitancy is relatively high among Chinese diabetes patients; education and specific guidelines are needed to overcome this resistance. For example, guidelines should specify the standard of vaccination for diabetes patients based on expert consensus.

This was the first study to use the HBM and multivariate logistic regression analysis to identify factors associated with SARS-CoV-2 vaccine hesitancy among diabetes patients in China. Social contextual factors related to patients’ reluctance to receive the SARS-CoV-2 vaccine included a high school education level or less. It is possible that patients with a lower education level are less likely to accept new things and were, thus, skeptical about the SARS-CoV-2 vaccine, which is supported by the results of a previous study in the general population [37]. In the future, health education initiatives should target diabetes patients with low education levels to increase the vaccination rate in this group.

Our HBM-based survey examined factors in five dimensions that could influence diabetes patients’ reluctance to receive the SARS-CoV-2 vaccine. First, false beliefs regarding the risk of SARS-CoV-2 infection among diabetes patients may have led to vaccine hesitancy: most patients in our study did not think that they were at high risk of infection but agreed that the prognosis of the disease was poor for patients with diabetes, in line with a previous report [38]. Second, most patients did not believe that vaccination would reduce their risk of SARS-CoV-2 infection, but agreed that it reduced the risk of transmission and that it was, therefore, beneficial to both themselves and others. This reflects the conflicted opinion of diabetes patients, who are uncomfortable with SARS-CoV-2 vaccination because of their physical condition but believe that vaccination of other diabetes patients can reduce the risk of virus transmission. Third, we found that concerns about vaccine safety (i.e., side effects) and efficacy were barriers to patients’ acceptance of the SARS-CoV-2 vaccine. This is consistent with the results of a nationwide online survey on SARS-CoV-2 vaccine hesitancy and requirement in China [31]. Fourth, the opinions and behaviors of people in their surroundings influenced patients’ willingness to be vaccinated, including the vaccination status of relatives and physicians’ view that the SARS-CoV-2 vaccine reduces the risk of infection. This is in accordance with an earlier report that diabetes patients’ vaccination behavior is susceptible to the opinions of others, and would only accept vaccination if the vaccine was administered to a large group of people [31]. Fifth, the promotion of the SARS-CoV-2 vaccine on social media influenced patients’ willingness to be vaccinated. It was demonstrated that receiving vaccination messages from trustworthy sources such as scientists or politicians could promote vaccine acceptance [39]. Thus, encouraging patients with diabetes to accept the SARS-CoV-2 vaccine through (social) media campaigns can increase the vaccination rate in this population [31].

This survey to the 273 diabetes patients with SARS-CoV-2 vaccine hesitancy revealed that the main reason for their reluctance was fear that the vaccine was unsafe and could have side effects. This is in line with findings from a previous study on attitudes toward influenza vaccination [40]. It is worth noting that the degree of concern among respondents was inversely related to their willingness to be vaccinated. Providing reports on the safety and side effects of the SARS-CoV-2 vaccine or firsthand accounts of patients with diabetes who have been vaccinated could allay these concerns. Moreover, the medium- and long-term effects are still unclear, and it is necessary for the health sector to further investigate the long-term immune response of diabetic patients to COVID-19 vaccination in the future, thereby improving the relevant cognition of diabetic patients as well as making more scientific decisions on vaccination behavior.

The results of this study have some practical implications. Given the factors that were identified as influencing diabetes patients’ willingness to receive the SARS-CoV-2 vaccine (e.g., education level), it is possible to increase the vaccination rate in this population through education and public awareness campaigns. Additionally, interventions can be implemented to address the factors contributing to vaccine hesitancy and increase confidence in the vaccine among diabetes patients.

A strength of this study is that it is the first to investigate factors associated with SARS-CoV-2 vaccine hesitancy in diabetes patients in a low- or middle-income country using the HBM. Our findings provide a reference for promoting vaccination against SARS-CoV-2 among diabetes patients in China and other low- and middle-income countries. However, our study also had some limitations. First, the sample was not representative of all diabetes patients in China because it was limited to one city, Changzhi. Moreover, this study was only conducted in a small sample size of 483 diabetic subjects; thus, in a future study, we will recruit participants from the general population in the same period as a control group to compare results. Additionally, we did not ask patients about their history of other vaccines, which could affect their SARS-CoV-2 vaccine status. Future studies need to consider this potentially important factor. Moreover, we condensed a five-point scale (strongly disagree; disagree; neutral; agree, and strongly agree) into a binary yes/no, and thus, we were losing potential information. We should use a more complex regression to explain all five options on the ordinal scale. Lastly, as the study was a cross-sectional survey, the causal relationship between predictors and outcome variables could not be determined.

## 5. Conclusions

In conclusion, we found that Chinese patients with diabetes have a higher rate of SARS-CoV-2 vaccine hesitancy than diabetes patients in other countries. Doubt regarding vaccine efficacy, lack of awareness of the risk of SARS-CoV-2 infection, and other factors, such as SARS-CoV-2 vaccine safety (side effects) and clinicians’ recommendations for SARS-CoV-2 vaccination, contribute to vaccine hesitancy. Appropriate education and interventions are needed to overcome Chinese diabetes patients’ reluctance to be vaccinated and reduce the risk of COVID-19–associated morbidity and mortality in this population.

## Figures and Tables

**Table 1:** Sociodemographic characteristics of the study population. (*n* = 483).

Characteristic	All Participants (*n* = 483) x ± S	Participants Willing to Receive SARS-CoV-2 Vaccine (*n* = 210) x ± S	Participants Hesitant to Receive SARS CoV-2 Vaccine (*n* = 273) x ± S	Hesitant vs. Willing Participants, c OR/t (95% CI)	*p*-Value
Sex					
Male	252 (52.2)	114 (54.3)	138 (50.5)	1.0	Ref
Female	231 (47.8)	96 (45.7)	135 (49.5)	0.86 (0.60–1.24)	0.42
Average age, years					
	56.43 ± 13.03	56.41 ± 12.37	56.45 ± 13.54	−0.03 (−2.39–2.31)	0.97
Age group, years					
18–39	50 (10.4)	19 (9.0)	31 (11.4)	1.0	Ref
40–49	78 (16.1)	43 (20.5)	35 (12.8)	1.21 (0.56–2.49)	0.61
50–59	143 (29.6)	59 (28.1)	84 (30.8)	0.60 (0.32–1.13)	0.11
60–69	132 (27.3)	55 (26.2)	77 (28.2)	1.05 (0.60–1.83)	0.86
≥70	80 (16.6)	34 (16.2)	46 (16.8)	1.03 (0.59–1.82)	0.91
Ethnicity					
Han	475 (98.3)	209 (99.5)	266 (97.4)	1.0	Ref
Other	8 (1.7)	1 (0.5)	7 (2.6)	0.18 (0.02–1.49)	0.11
Education level					
Below high school	288 (59.6)	114 (54.3)	174 (63.7)	1.0	Ref
High school	95 (19.7)	45 (21.4)	50 (18.3)	1.59 (1.01–2.51)	0.05
College and above	100 (20.7)	51 (24.3)	49 (17.9)	1.16 (0.66–2.03)	0.61
Marital status					
Unmarried, divorced, separated, or widowed	59 (12.2)	22 (10.5)	37 (13.6)	1.0	Ref
Married or cohabitating	424 (87.8)	188 (89.5)	236 (86.4)	1.34 (0.76–2.35)	0.31
Occupation type					
Farmer	210 (43.5)	92 (43.8)	118 (43.2)	1.0	Ref
Public institution personnel	90 (18.6)	49 (23.3)	41 (15.0)	0.65 (0.40–1.07)	0.09
Business staff	55 (11.4)	17 (8.1)	38 (13.9)	1.74 (0.93–3.28)	0.08
Worker	74 (15.3)	32 (15.2)	42 (15.4)	1.02 (0.60–1.75)	0.93
Transportation staff	29 (6.0)	11 (5.2)	18 (6.6)	1.28 (0.57–2.83)	0.55
Other	25 (5.2)	9 (4.3)	16 (5.9)	1.39 (0.59–3.28)	0.46
Residence					
Urban	267 (55.3)	115(54.8)	152 (55.7)	1.0	Ref
Rural	216 (44.7)	95 (45.2)	121 (44.3)	1.04 (0.72–1.49)	0.84
Monthly personal income (Chinese yuan ^†^)					
<2000	205 (42.4)	85 (40.5)	120 (44.0)	1.0	Ref
2000–3499	142 (29.4)	58 (27.6)	84 (30.8)	0.92 (0.47–1.81)	0.82
3500–4999	93 (19.3)	50 (23.8)	43 (15.8)	0.95 (0.47–1.90)	0.88
≥5000	43 (8.9)	17 (8.1)	26 (9.5)	0.56 (0.27–1.17)	0.13
Type of insurance					
Urban worker	228 (47.2)	108 (51.4)	120 (44.0)	1.0	Ref
Urban residents	113 (23.4)	41 (19.5)	72 (26.4)	0.84 (0.55–1.28)	0.41
New Rural Cooperative Medical Scheme	142 (29.4)	61 (29.0)	81 (29.7)	1.32 (0.80-2.20)	0.28

Data are shown as n (%). ^†^ A currency exchange rate of 1 Chinese yuan = US $0.16 was applied. Abbreviations: CI, confidence interval; cOR, crude odds ratio; Ref, reference; SARS-CoV-2, severe acute respiratory syndrome-associated coronavirus 2.

**Table 2:** Lifestyle and health conditions of study participants.

Condition	All Participants (*n* = 483) x ± S	Participants Willing to Receive SARS-CoV-2 Vaccine x ± S (*n* = 210)	Participants Hesitant to Receive SARS-CoV-2 Vaccine x ± S (*n* = 273)	Hesitant vs. Willing Participants, c OR/t (95% CI)	*p*-Value
Current smoker					
No	145 (30.0)	61 (29.0)	84 (30.8)	1.0	Ref
Yes	338 (70.0)	149 (71.0)	189 (69.2)	1.10 (0.73–1.61)	0.68
Current drinker					
No	121 (25.1)	52 (24.8)	69 (25.3)	1.0	Ref
Yes	362 (74.9)	158 (75.2)	204 (74.7)	1.03 (0.68–1.56)	0.90
Average self-reported BMI, kg/m^2^					
	25.27 ± 4.85	25.16 ± 5.28	25.36 ± 4.50	−0.44 (−1.07–0.68)	0.66
Self–reported BMI, kg/m^2^					
<18.5	55 (11.4)	23 (11.0)	32 (11.7)	1.0	Ref
18.5–23.9	186 (38.5)	88 (41.9)	98 (35.9)	0.86 (0.43–1.73)	0.68
24.0–27.9	161 (33.3)	68 (32.4)	93 (34.1)	0.69 (0.41–1.18)	0.17
≥28	81 (16.8)	31 (14.8)	50 (18.3)	0.85 (0.49–1.46)	0.55
Other chronic diseases					
No	67 (13.9)	35 (16.7)	32 (11.7)	1.0	Ref
Yes	416 (86.1)	175 (83.3)	241 (88.3)	0.66 (0.40–1.11)	0.12
Diabetes complications					
No	159 (32.9)	73 (34.8)	86 (31.5)	1.0	Ref
Yes	324 (67.1)	137 (65.2)	187 (68.5)	0.86 (0.59–1.27)	0.45
Controlled blood glucose level					
No	262 (54.2)	109 (51.9)	153 (56.0)	1.0	Ref
Yes	221 (45.8)	101 (48.1)	120 (44.0)	1.18 (0.82–1.70)	0.37
Family history of diabetes					
No	173 (35.8)	74 (35.2)	99 (36.3)	1.0	Ref
Yes	310 (64.2)	136 (64.8)	174 (63.7)	1.05 (0.72–1.52)	0.82
Average time since diabetes diagnosis, years					
	7.72 ± 7.29	7.58 ± 7.49	7.83 ± 7.14	−0.37 (−1.57–1.07)	0.71
Time since diabetes diagnosis, years					
≤1	141 (29.2)	63 (30.0)	78 (28.6)	1.0	Ref
2–10	196 (40.6)	84 (40.0)	112 (41.0)	0.94 (0.59–1.50)	0.79
>10	146 (30.2)	63 (30.0)	83 (30.4)	1.01 (0.66–1.56)	0.96
Average fasting blood glucose at most recent testing, mmol/L					
	8.67 ± 4.99	8.68 ± 6.75	8.66 ± 3.02	0.04 (−0.89–0.92)	0.97
Fasting blood glucose at most recent testing, mmol/L					
<7	169 (35.0)	77 (36.7)	92 (33.7)	1.0	Ref
7–13.9	276 (57.1)	120 (57.1)	156 (57.1)	0.62 (0.30–1.30)	0.21
>13.9	38 (7.9)	13 (6.2)	25 (9.2)	0.68 (0.33–1.38)	0.28
Average postprandial blood glucose at most recent testing, mmol/L					
	12.74 ± 11.60	12.32 ± 9.81	13.06 ± 12.83	−0.69 (−2.84–1.36)	0.49
Postprandial blood glucose at most recent testing, mmol/L					
<10	159 (32.9)	68 (32.4)	91 (33.3)	1.0	Ref
10–11.1	87 (18.0)	46 (21.9)	41 (15.0)	0.91 (0.61–1.37)	0.65
>11.1	237 (49.1)	96 (45.7)	141 (51.6)	0.66 (0.35–1.24)	0.19

Data are shown as *n* (%). Abbreviations: BMI, body mass index; CI, confidence interval; cOR, crude odds ratio; Ref, reference; SARS-CoV-2, severe acute respiratory syndrome-associated coronavirus 2.

**Table 3:** Willingness to receive the SARS-CoV-2 vaccine among study participants and variables included in the health belief model.

Variable	All Participants (*n* = 483)	Participants Willing to Receive SARS-CoV-2 Vaccine (*n* = 210)	Participants Hesitant to Receive SARS-CoV-2 Vaccine (*n* = 273)	Hesitant vs. Willing Participant, c OR (95% CI)	*p*-Value
Willing to receive SARS-CoV-2 vaccine					
No (unwilling or unsure)	273 (56.4)	0 (0)	273 (100)	N/A	N/A
Yes (willing)	210 (43.6)	210 (100)	0 (0)	N/A	N/A
Perceived susceptibility					
Risk of acquiring SARS-CoV-2 is high					
No (strongly disagree or disagree or neutral)	371 (76.8)	157 (74.8)	214 (78.4)	1.0	Ref
Yes (agree or strongly agree)	112 (23.2)	53 (25.2)	59 (21.6)	1.22 (0.80–1.87)	0.35
Perceived severity					
SARS-CoV-2 syndrome is severe					
No (strongly disagree or disagree or neutral)	329 (68.1)	177 (84.3)	152 (55.7)	1.0	Ref
Yes (agree or strongly agree)	154 (31.9)	33 (15.7)	121 (44.3)	4.27 (2.75–6.64)	<0.001
Perceived benefits					
Vaccination reduces the risk of infection					
Yes (agree or strongly agree)	164 (34.0)	89 (42.4)	75 (27.5)	1.0	Ref
No (strongly disagree or disagree or neutral)	319 (66.0)	121 (57.6)	198 (72.5)	1.94 (1.33–2.84)	0.001
Vaccination reduces the risk of transmission to other people					
Yes (agree or very agree)	297 (61.5)	149 (71.0)	148 (54.2)	1.0	Ref
No (strongly disagree or disagree or neutral)	186 (38.5)	61 (29.0)	125 (45.8)	2.06 (1.41–3.02)	<0.001
Vaccination is good for myself and others					
Yes (agree or strongly agree)	463 (95.9)	202 (96.2)	261 (95.6)	1.0	Ref
No (strongly disagree or disagree or neutral)	20 (4.1)	8 (3.8)	12 (4.4)	1.16 (0.47–2.89)	0.75
Perceived barriers					
As a person with diabetes, I worry about the safety of the SARS-CoV-2 vaccine					
No (strongly disagree or disagree or neutral)	146 (30.2)	95 (45.2)	51 (18.7)	1.0	Ref
Yes (agree or strongly agree)	337 (69.8)	115 (54.8)	222 (81.3)	3.60 (2.39–5.41)	<0.001
I am worried about side effects of vaccination					
No (strongly disagree or disagree or neutral)	200 (41.4)	116 (55.2)	84 (30.8)	1.0	Ref
Yes (agree or strongly agree)	283 (58.6)	94 (44.8)	189 (69.2)	2.77 (1.91–4.04)	<0.001
Action clues					
Relatives’ vaccination status will affect my vaccination behavior					
Yes (agree or strongly agree)	85 (17.6)	52 (24.8)	33 (12.1)	1.0	Ref
No (strongly disagree or disagree or neutral)	398 (82.4)	158 (75.2)	240 (87.9)	2.40 (1.48–3.87)	<0.001
I accept physicians’ view that vaccination can reduce the risk of infection					
Yes (agree or strongly agree)	253 (52.4)	150 (71.4)	103 (37.7)	1.0	Ref
No (strongly disagree or disagree or neutral)	230 (47.6)	60 (28.6)	170 (62.3)	4.13 (2.80–6.07)	<0.001
I have received advice on vaccination from the internet/media					
Yes (agree or strongly agree)	233 (48.2)	124 (59.0)	109 (39.9)	1.0	Ref
No (strongly disagree or disagree or neutral)	250 (51.8)	86 (41.0)	164 (60.1)	2.17 (1.50–3.13)	<0.001

Data are shown as n (%). Abbreviations: CI, confidence interval; cOR, crude odds ratio; N/A, not applicable; Ref, reference; SARS-CoV-2, severe acute respiratory syndrome-associated coronavirus 2.

**Table 4:** Multivariate model of the factors associated with diabetes patients’ hesitation to receive the SARS-CoV-2 vaccine.

Variable	a OR (95%CI) ^†^	*p*-Value
Perceived susceptibility		
Risk of acquiring SARS-CoV-2 is high		
No (strongly disagree or disagree or neutral)	1.0	Ref
Yes (agree or strongly agree)	0.79 (0.47–1.32)	0.36
Perceived severity		
SARS-CoV-2 syndrome is severe		
No (strongly disagree or disagree or neutral)	1.0	Ref
Yes (agree or strongly agree)	3.90 (2.36–6.42)	<0.001
Perceived benefits		
Vaccination reduces the risk of infection		
Yes (agree or strongly agree)	1.0	Ref
No (strongly disagree or disagree or neutral)	0.65 (0.41–1.03)	0.07
Vaccination reduces the risk of transmission to other people		
Yes (agree or very agree)	1.0	Ref
No (strongly disagree or disagree or neutral)	1.22 (0.77–1.94)	0.40
Vaccination is good for myself and others		
Yes (agree or strongly agree)	1.0	Ref
No (strongly disagree or disagree or neutral)	1.09 (0.40–2.99)	0.87
Perceived barriers		
As a person with diabetes, I worry about the safety of the SARS-CoV-2 vaccine		
No (strongly disagree or disagree or neutral)	1.0	Ref
Yes (agree or strongly agree)	3.05 (1.89–4.91)	<0.001
I am worried about side effects of vaccination		
No (strongly disagree or disagree or neutral)	1.0	Ref
Yes (agree or strongly agree)	0.71 (0.41–1.25)	0.24
Action clues		
Relatives’ vaccination status will affect my vaccination behavior		
Yes (agree or strongly agree)	1.0	Ref
No (strongly disagree or disagree or neutral)	2.43 (1.39–4.25)	0.002
I accept physicians’ view that vaccination can reduce the risk of infection		
Yes (agree or strongly agree)	1.0	Ref
No (strongly disagree or disagree or neutral)	2.25 (1.28–3.95)	0.005
I have received advice on vaccination from the internet/media		
Yes (agree or strongly agree)	1.0	Ref
No (strongly disagree or disagree or neutral)	1.29 (0.83–2.00)	0.26

^†^ aOR was obtained by fitting a single logistic regression model involving an independent variable of interest and all background variables listed in Table 1 and Table 2 with *p* < 0.10 in the univariate analysis. Abbreviations: aOR, adjusted odds ratio; CI, confidence interval; Ref, reference; SARS-CoV-2, severe acute respiratory syndrome-associated coronavirus 2.

## Data Availability

Not applicable.
